# MENTAL AND PHYSICAL HEALTH SEQUELAE OF BEREAVEMENT IN OLDER ADULTS: U.S. HEATH AND RETIREMENT STUDY ANALYSIS

**DOI:** 10.1093/geroni/igz038.1260

**Published:** 2019-11-08

**Authors:** Benjamin Domingue, Laramie Duncan, Amal Harrati, Daniel Belsky

**Affiliations:** 1 Stanford University Graduate School of Education, Stanford, California, United States; 2 Psychiatry and Behavioral Sciences, Stanford, California, United States; 3 Primary Care & Population Health, Stanford, California, United States; 4 Epidemiology, New York, New York, United States

## Abstract

Death of a spouse (bereavement) is associated with poor mental and physical health outcomes in older adults. But it is unknown how mental- and physical-health sequelae of bereavement are related and the clinical significance of bereavement-related depression has been questioned. We analyzed US Health and Retirement Study (HRS) data tracking mental and physical health of 36,034 older adults during 1992-2016. Post-bereavement data were available for N=4,985 participants with recorded date of spousal death. We analyzed longitudinal repeated-measures data on survivors’ depression, disease, disability, and mortality. Bereavement effects on depression were immediate, but short-lived, attenuating within the year. In contrast, bereavement effects on physical health and mortality persisted over follow-up. Critically, the magnitude of short-lived effects on depression correlated with the magnitude of longer-lasting effects on disease, disability, and mortality. Results reveal connections between mental and physical health and aging and suggest bereavement-related depression as a biomarker of enduring health risk.

